# High-Pressure Processing Alters Biofilm Persistence and Virulence Gene Expression in *Listeria monocytogenes* Strains

**DOI:** 10.3390/ijms27125366

**Published:** 2026-06-14

**Authors:** Patryk Adamski, Arkadiusz Józef Zakrzewski, Anna Zadernowska, Wioleta Chajęcka-Wierzchowska

**Affiliations:** Department of Food Microbiology, Meat Technology and Chemistry, Faculty of Food Science, University of Warmia and Mazury, Plac Cieszyński 1, 10-726 Olsztyn, Poland; anna.zadernowska@uwm.edu.pl (A.Z.); wioleta.chajecka@uwm.edu.pl (W.C.-W.)

**Keywords:** *L. monocytogenes*, stress response, food-contact surfaces, biofilms

## Abstract

*Listeria monocytogenes* is a persistent foodborne pathogen capable of forming biofilms and surviving in food-processing environments. This study investigated the impact of high-pressure processing (HPP) at 200 and 400 MPa/5 min on biofilm viability, biomass, and expression of nine virulence-associated genes in *L. monocytogenes* strains (*n* = 6) belonging to the serogroups IIa (LM8, LM40, LM41) and IVb (LM14, LM47, LM48). The pressure levels applied were selected to represent sublethal HPP conditions (below 600 MPa) that allowed the survival of the strains and thus enabled the investigation of adaptive responses in cells that escape complete inactivation. Biofilms were cultivated on stainless-steel 304, polyethylene terephthalate, and polypropylene coupons under static conditions at 25 °C for 72 h and 168 h. Biofilm viability [log_10_(CFU/cm^2^)] was assessed by plate count method and biomass quantified via the biofilm production index (BPI). The cultures were subjected to HPP treatment and their ability to form biofilms was re-evaluated. HPP significantly (*p* < 0.05) reduced biofilm viability and biomass on all types of surfaces tested. Gene expression analysis revealed a pressure-dependent (*p* < 0.05) modulation of *flaA* and *sigB*, while other virulence genes (*agrA*, *agrC*, *actA*, *prfA*, *hly*, *inlB*, and *degU*) were generally downregulated (gene expression ratio values below 1). Serogroup IVb strains exhibited enhanced stress responses and lower biofilm survival on polyethylene terephthalate and polypropylene surfaces. These findings demonstrate that HPP modulates both phenotypic and genotypic traits linked to *L. monocytogenes* persistence, emphasizing the need to optimize pressure parameters and surface materials to prevent biofilm formation in HPP-treated food systems.

## 1. Introduction

*Listeria monocytogenes* is a Gram-positive, non-spore-forming bacterium that is responsible for listeriosis, a foodborne infection that predominantly affects individuals with compromised immune systems and those over the age of 64 years [1]. As demonstrated in the report by the European Food Safety Authority (EFSA) and the European Centre of Disease Prevention and Control (ECDC) in 2024, there has been an increase in the number of confirmed cases of listeriosis reported in the European Union (EU) [2,3].

*L. monocytogenes* is considered among the most significant pathogens present in food-manufacturing facilities. Research has demonstrated that production environments can be colonized over extended periods by *L. monocytogenes* strains, with certain sequence types persisting on surfaces and in facilities across months or years [3,4]. The ability of these strains to survive cleaning and disinfection procedures is largely attributable to their capacity to form biofilms (BFCs), which confer increased tolerance to biocides under conditions typical of food-processing environments [5,6]. Biofilms are highly structured bacterial consortia that irreversibly attach to surfaces and become encased in a self-secreted extracellular polymeric matrix [7,8].

*L. monocytogenes* exhibits considerable genetic diversity. It is classified into four major evolutionary lineages, four PCR-based serogroups, and 14 recognized serotypes. Moreover, this species can be further differentiated into clonal complexes (CCs) and sequence types (STs) using multilocus sequence typing (MLST) [9]. The biofilm-forming ability of *L. monocytogenes* is also associated with its serogroup classification, with clear differences observed between serogroups IIa and IVb. Strains belonging to serogroup IIa are commonly isolated from food and food-processing environments and are generally characterized by a higher capacity for adhesion and robust biofilm formation. This enhanced biofilm phenotype is linked to their adaptation to environmental stresses, persistence on abiotic surfaces, and the presence of genetic determinants supporting survival under suboptimal conditions [10,11]. In contrast, serogroup IVb is more frequently associated with clinical cases and outbreaks of listeriosis. Although IVb strains possess a high virulence potential, they typically exhibit a lower biofilm-forming capacity and reduced persistence in food-processing environments compared to IIa isolates [12]. These differences suggest a trade-off between virulence and environmental fitness, where serogroup IIa strains are better adapted for survival and biofilm formation in industrial settings, while serogroup IVb strains are more specialized for host infection.

Bacteria encounter numerous stressors both in natural ecosystems and during host colonization. Within food production systems, one notable stressor is the application of high-pressure processing (HPP). This non-thermal preservation method effectively inactivates a broad spectrum of microorganisms, thereby significantly prolonging the shelf life of food products [13,14]. HPP remains effective against both serogroups, although strain-dependent variability can occur. Pressures in the range of 300–400 MPa generally induce sublethal injury in *L. monocytogenes* cells, whereas treatments in the range of 500–600 MPa are considered lethal, resulting in substantial reductions in viable populations [15]. Pressures ≥ 600 MPa are typically required to ensure near-complete inactivation, due to irreversible damage to cellular membranes, protein structures, and essential metabolic functions [16,17].

Apart from food-processing environments, *L. monocytogenes* can also form biofilms on food packaging materials [18,19]. Moreover, fruit juices are among the most common food products preserved by HPP [15] and plastic packaging is a frequent site of biofilm formation [20,21], where *L. monocytogenes* can adhere to and develop on bottle walls and bottoms. Other studies have shown that *L. monocytogenes* cells can recover after HPP treatment and restore their metabolic activity, while HPP can simultaneously influence the expression of virulence genes in strains isolated from food or food-processing environments [22,23,24].

In light of these findings, improperly selected pressure parameters or the presence of highly baroresistant strains may potentially lead to biofilm formation inside the packaging materials of products previously subjected to HPP. While numerous studies have examined the inactivation of *L. monocytogenes* by HPP treatment, few have simultaneously explored how sublethal HPP stress modulates both the bacterium’s ability to form biofilms and the expression of virulence-related genes. Understanding these concurrent phenotypic and genotypic responses is crucial for evaluating the adaptive potential of *L. monocytogenes* under industrial preservation conditions. Therefore, the aim of this study was to evaluate the pressure-dependent modulation of the biofilm-forming capacity and virulence gene expression of *L. monocytogenes* strains exposed to HPP. The experiments were conducted on surfaces commonly present in the food chain to provide a realistic assessment of potential post-HPP survival and biofilm development. To the best of our knowledge, this is the first study to simultaneously evaluate pressure-dependent changes in biofilm-forming ability and virulence gene expression of *L. monocytogenes* following sublethal HPP exposure on food-chain-relevant surfaces.

## 2. Results

### 2.1. Gene Expression

**Figure 1:**
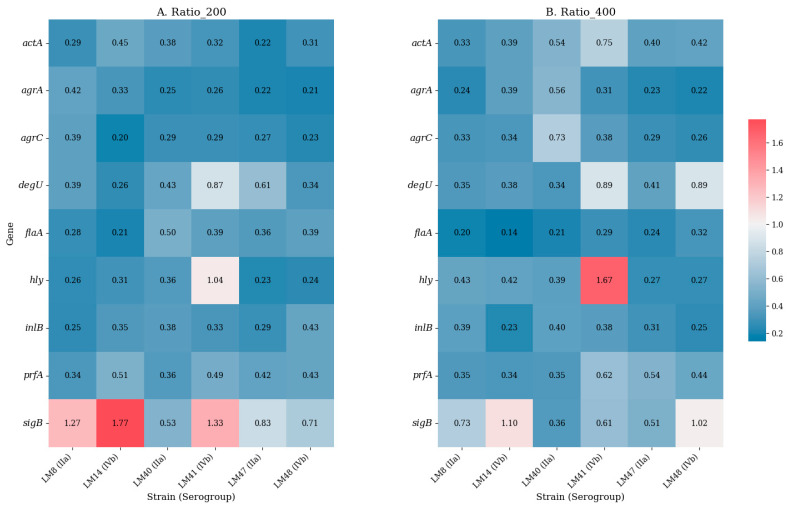
Heatmaps showing gene expression ratios across *L. monocytogenes* strains after HPP treatments: (**A**) 200 MPa/5 min; (**B**) 400 MPa/5 min. Scale: Higher values indicate increased gene expression ratio, where 1 denotes baseline expression, 0.5 (dark blue) indicates substantial underexpression.

Among the analyzed genes, *flaA* and *sigB* showed statistically significant (*p* < 0.05) differences in expression between exposure to 200 MPa and 400 MPa/5 min, indicating pressure-dependent regulation. All other genes, *agrA*, *agrC*, *actA*, *prfA*, *hly*, *inlB*, and *degU*, did not exhibit statistically significant changes (*p* > 0.05) in expression between parameters. In most cases, gene expression levels indicated underexpression regardless of exposure to HPP pressure (Figure 1).

### 2.2. Biofilm Viability

The application of HPP treatment had a significant effect (T: Kruskal–Wallis; *p* < 0.05) on biofilm formation, as HPP-treated cells formed biofilms, resulting in markedly reduced cell counts [log_10_(CFU/cm^2^)] of the tested *L. monocytogenes* strains on all examined surfaces (SS304, PET, PP). Under control conditions, after 72 h, log_10_(CFU/cm^2^) values exceeded 7.0, indicating a strong ability of the strains to form biofilms. After 168 h, only a slight decrease was observed for mature biofilms on PP coupons (6.60 ± 0.30). HPP treatment at 200 MPa/5 min caused a moderate reduction, whereas 400 MPa/5 min resulted in a substantial decrease in cell counts, down to ~5 log_10_(CFU/cm^2^) (Table 1).

Additionally, statistically significant differences between serogroups IIa and IVb were observed across different surfaces and incubation times. Notably, mature biofilms (168 h) formed on PET and PP surfaces showed reduced viable cell counts in IVb strains compared to IIa (*p* = 0.020 and *p* = 0.013, respectively). A significant difference was also detected on PET after 72 h (*p* = 0.049), indicating that serogroup IVb strains may exhibit lower survival even during the final phase of young biofilms on PET and PP. The viable cell counts [log_10_(CFU/cm^2^)] for individual strains are listed in Table S2.

### 2.3. Biofilm Biomass

**Figure 2:**
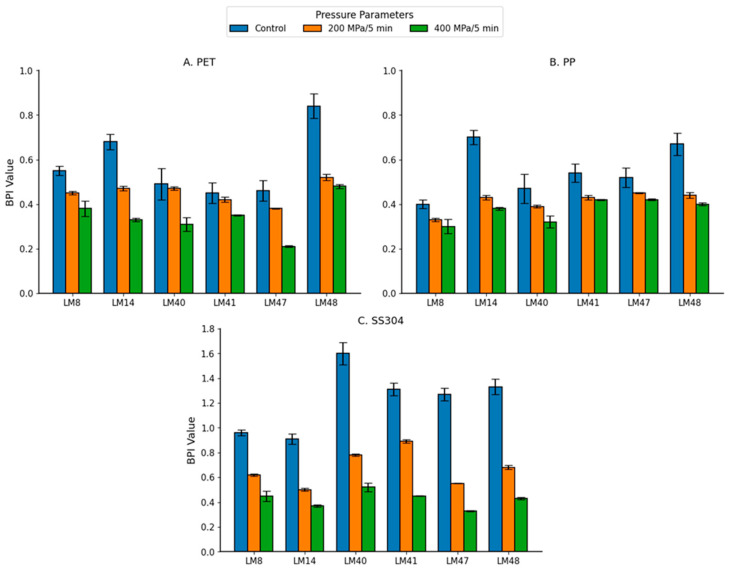
BPI values [mean ± SD] of *L. monocytogenes* strains (*n* = 6) on three surface types following HPP treatment at 200 MPa and 400 MPa/5 min. Control samples represent untreated strains. Note the distinct y-axis scaling for SS304 (0–1.8) versus PET and PP (0–1.0), reflecting higher baseline biofilm integrity on SS304.

Based on the comparative analysis of biofilm biomass formed by tested *L. monocytogenes* strains on PET, PP, and SS304 surfaces under control conditions and after HPP treatments (200 MPa/5 min and 400 MPa/5 min), statistically significant differences were observed. Under control conditions, SS304 supported the highest biofilm biomass values (1.23 ± 0.27) (Figure 2), significantly greater than PET (0.58 ± 0.15) (Figure 2) and PP (0.55 ± 0.11) (Figure 2), with ANOVA confirming a surface effect (*p* < 0.000001). Post hoc Tukey HSD tests revealed significant pairwise differences between SS304 and both PET (*p* < 0.000001) and PP (*p* < 0.000001). After treatment at 200 MPa/5 min, biofilm biomass decreased across all surfaces, yet SS304 remained significantly higher (0.67 ± 0.14) than PET (0.45 ± 0.05) and PP (0.41 ± 0.05), with ANOVA *p* = 0.0013 and Tukey comparisons confirming significance for SS304 vs. PET (*p* < 0.003) and SS304 vs. PP (*p* < 0.001). At 400 MPa/5 min, biofilm levels were lowest overall (PET: 0.34 ± 0.08; PP: 0.37 ± 0.05; SS304: 0.42 ± 0.07) and ANOVA indicated a marginally significant surface effect (*p* = 0.0482). These results demonstrate that SS304 consistently supports higher biofilm biomass production of *L. monocytogenes* strains at 25 °C/24 h.

## 3. Discussion

The present study demonstrates that exposure of *L. monocytogenes* to HPP treatments significantly affects both biofilm formation and the expression of selected virulence-associated genes. Increasing pressure parameters from 200 MPa to 400 MPa led to a reduction in biofilm biomass and cell viability across all tested surfaces (SS304, PET, and PP), indicating a clear pressure-dependent reduction in biofilm biomass and cell viability.

Based on other studies, transcriptional responses to HPP are highly strain- and serotype-dependent. Previous studies demonstrated that virulence and stress-related genes may be overexpressed or suppressed depending on the strain and serotype [22,23,25]. Such ecological specialization may influence the overall response of *L. monocytogenes* to high-pressure stress. Serogroup IVb strains, belonging to lineage I, are predominantly associated with clinical cases and outbreaks, whereas serogroup IIa strains (lineage II) are more frequently isolated from food and food-processing environments and are considered better adapted to environmental persistence [3,7]. These differences suggest that the two groups may exhibit distinct responses to technological stresses such as HPP.

In this context, IVb strains demonstrated a measurable stress response; however, this was not reflected in improved survival within biofilms. This observation indicates that the relationship between stress response and survival under HPP conditions is not straightforward and may depend on multiple factors beyond transcriptional activity alone. High-pressure processing induces a range of cellular effects, including membrane disruption, protein denaturation, and impairment of cellular functions, which collectively determine the ability of bacteria to survive and recover [16,17]. Although no statistically significant differences were observed between serogroups IIa (LM8, LM40, LM41) and IVb (LM14, LM47, LM48), gene expression ratios revealed consistent trends. Gene *sigB* was more strongly activated in IVb strains at 200 MPa (1.10) and 400 MPa (0.88) compared to IIa (0.71 and 0.57), reflecting enhanced general stress response in IVb, consistent with the findings of [25]. *prfA* expression was slightly elevated in IVb (0.45 at 200 MPa) versus IIa (0.40), aligning with reports of virulence activation in IVb strains [23] (Pérez-Baltar et al., 2021). Next, *hly*—the hemolysin gene [26]—followed a similar pattern: IVb strains maintained moderate expression (0.27–0.30), whereas LM41 (IIa) exhibited spikes at 200 and 400 MPa (1.04 and 1.67, respectively), highlighting intra-serogroup variability. *inlB*, which encodes cell surface invasins [26], remained relatively stable (0.25–0.43), with no clear serogroup dependence, while *actA*, which encodes actin-polymerizing protein [27], varied modestly, peaking in LM41 (IIa, 0.75 at 400 MPa) but remaining below 0.42 in IVb strains, suggesting differential pressure sensitivity. Quorum-sensing regulators *agrA* and *agrC* were mildly repressed in IVb (0.22–0.29), consistent with post-HPP attenuation of cell–cell signaling [7], whereas IIa strains (LM40, LM41) showed higher *agrA* expression (up to 0.56 at 400 MPa), possibly reflecting enhanced recovery dynamics. *flaA*, associated with motility, was low and uniform (0.2–0.5), supporting its role in repair rather than virulence [28]. Finally, *degU*, a key biofilm regulator [28], was moderately downregulated in LM41 (IIa) and LM48 (IVb) at 400 MPa (0.89), suggesting a pressure-induced attenuation of biofilm formation pathways. Furthermore, according to the study by Gueriri et al. [28], Δ*degU* mutants in which the entire *degU* gene was deleted inactivated the expression of the *flaA* gene. Therefore, low expression of the *degU* gene may translate into the low expression of the *flaA* gene observed by us. Furthermore, Δ*degU* mutants formed significantly weaker biofilms than the parental strains [28].

At the molecular level, HPP induced significant modulation of *flaA* and *sigB* expression, while most other virulence genes, including *actA*, *inlB*, *prfA*, and *hly*, were downregulated. Upregulation of *sigB* at higher pressures reflects the activation of the general stress response regulon, supporting survival under adverse environmental conditions such as low pH or high osmotic pressure, whereas changes in *flaA* expression likely influence motility and adhesion, key factors in initial biofilm formation [28]. It was observed that the direction of transcriptomic response in the *sigB* gene varied among the strains examined. It was observed that certain strains exhibited standard expression, as evidenced by a gene expression ratio approximating 1. In contrast, other strains demonstrated moderate downregulation when subjected to a pressure of 200 MPa. At 400 MPa, two strains (LM14 and LM48) demonstrated standard expression. The remaining samples exhibited underexpression, characterized by a decline in gene expression relative to the 200 MPa. The results obtained appear to corroborate the findings presented by Bowman et al. [25] suggesting a gradual decline in *sigB* gene expression with increasing HPP pressure. The observed downregulation of other virulence genes may indicate a transient suppression of energy-demanding virulence mechanisms, allowing cells to focus on structural repair under stress [16]. However, it remains difficult to unequivocally attribute the observed reduction in biofilm formation [log_10_(CFU/cm^2^)] solely to changes in *sigB* and *flaA* expression (200 MPa/5 min against 400 MPa/5 min). This is because sublethal HPP exposure resulted in the underexpression of a broader set of genes associated with stress response and biofilm development, suggesting that the observed phenotype likely arises from a cumulative, multifactorial regulatory effect rather than from the modulation of individual genes alone. Consequently, although the detected differences in gene expression were statistically significant, their biological relevance should be interpreted with caution, as they may reflect a global stress-induced transcriptional reprogramming rather than direct, gene-specific control of biofilm formation. Collectively, these patterns suggest that HPP shifts bacterial physiology toward survival rather than virulence or colonization.

Surface characteristics significantly influenced biofilm formation. SS304 consistently supported higher biofilm biomass production, consistent with the known affinity of *L. monocytogenes* for hydrophilic, rough surfaces [6]. Notably, the inhibitory effect of HPP was more pronounced on PET and PP, indicating that material properties modulate the bacterium’s ability to withstand pressure and reattach. Differences between BPIs and viable cell counts [log_10_(CFU/cm^2^)] reflect the distinct aspects these methods measure. BPI quantifies total biofilm biomass, including live and dead cells as well as extracellular polymeric substances (EPSs), whereas viable cell counts consider only cells capable of replication, explaining discrepancies between the two approaches [29]. It should also be noted that BPI values were obtained after 24 h of incubation, representing the stage of early biofilm formation, while viable cell counts were determined after 72 h and 168 h. Consequently, BPI determination at later stages would not accurately represent biomass formation dynamics, as the number of dead cells would increase [29]. On SS304, the highest BPI values likely reflect strong adhesion and dense biofilm architecture rather than maximal cell viability. Dense, multilayered biofilms can further limit oxygen and nutrient diffusion to deeper layers, reducing viability in part of the population [29]. Consequently, high BPI values do not necessarily correlate with high numbers of viable cells, a consideration critical for interpreting quantitative biofilm assessments [29].

The ability of *L. monocytogenes* to regenerate after exposure to HPP, especially at 400 MPa/5 min, which is commonly used in the food industry, may be of significant importance for food safety [16,22,25]. In future studies, it would be valuable to include transcriptomic analysis of cells after successive passages to determine whether the observed changes in gene expression are transient or permanent, including in the analysis other serogroups and a larger number of strains. From an industrial perspective, the obtained data indicate the need to adjust HPP parameters not only according to the type of product but also to the properties of surfaces that microorganisms may contact after processing. Although SS304 is not used as a packaging material, it represents a standard contact surface in food-processing facilities, present on production lines, work tables, mixers, and packaging equipment [6,10]. The results confirm that SS304 promotes the formation of biofilms with the highest biomass, which may increase the risk of cross-contamination. *L. monocytogenes* biofilms on such surfaces can serve as persistent reservoirs of the pathogen, resistant to standard cleaning and disinfection procedures [6,29], and their presence may lead to secondary contamination of products even after HPP treatment. In contrast, polymeric packaging materials, such as PET and PP, may not ensure complete elimination of cells under sublethal conditions. Therefore, the design of packaging systems and the selection of HPP parameters should consider both the properties of contact materials and their potential interaction with high-pressure technology to minimize the risk of pathogen survival and recovery.

Despite providing novel insights into the effects of sublethal HPP exposure on biofilm formation and virulence gene expression in *L. monocytogenes* strains, this study has limitations that should be acknowledged. One important limitation is the use of only six strains, which restricts the generalizability of the findings; including a larger number of isolates representing additional serogroups would strengthen the robustness of future analyses. Although surface roughness and hydrophobicity (contact angle) were not measured in this study, we acknowledge that these parameters can influence *L. monocytogenes* adhesion and early biofilm development. Including such physicochemical characterization in future work would strengthen the interpretation of material dependent effects. The experimental design focused on the expression of a selected set of virulence-associated genes; therefore, the broader regulatory networks and molecular mechanisms underlying the observed responses to HPP remain unresolved. In particular, the potential involvement of global stress regulators, signal transduction pathways, and protein–protein interaction networks associated with pressure-induced stress responses was not investigated. Consequently, the mechanistic links between transcriptional changes and the observed biofilm phenotypes require further clarification. Future studies employing comprehensive approaches such as transcriptomics, proteomics, or metabolomics could provide deeper insight into the regulatory mechanisms governing the adaptive response and persistence strategies of *L. monocytogenes* under sublethal high-pressure stress.

## 4. Materials and Methods

### 4.1. Strains

Six *L. monocytogenes* strains were used in this study. The strains originated from the culture collection of the Department of Food Microbiology, Meat Technology and Chemistry, University of Warmia and Mazury in Olsztyn. The examined strains belonged to serogroups IIa (LM8, LM40, LM41) and IVb (LM14, LM47, LM48). A detailed characterization of the strains is provided in previously published papers by Zakrzewski et al. [30,31]. The whole-genome sequences of the analyzed strains have been deposited in the NCBI database under BioProject accession numbers PRJNA860799 and PRJNA945026. All strains were stored in Microbank^TM^ cryovials (PRO-LAB diagnostic, Richmond Hill, ON, Canada) at −80 °C. Before use, the cultures were revived by streaking on Tryptic Soy Agar (TSA; Merck, Darmstadt, Germany) and incubated at 37 °C for 24 h.

### 4.2. HPP Treatment and Recovery

Each tested strain of *L. monocytogenes* was subjected to HPP treatment as described in previous work by the authors: Adamski et al. [32]. Briefly, 10 mL of a 24 h TSB culture was transferred into low-density polyethylene bottles and exposed to 200 and 400 MPa/5 min at 20 ± 3 °C using a U4040 high-pressure unit (IWC PAN, Warsaw, Poland). The system reached the target pressure with a ramp rate of 300 MPa/min, and pressure release was completed in under 5 s. Non-pressurized samples served as controls. The pressure levels applied were intentionally selected to represent sublethal HPP conditions (below 600 MPa). This approach enabled the investigation of adaptive phenotypic and genotypic responses in cells that escape lethal treatment, which may occur in practice due to process variability, product characteristics. According to the authors’ previous work (Adamski et al. [32]), the strains tested maintained high viability (~10^8^ CFU/mL) after HPP treatment at 200 MPa/5 min. At the same time, a pressure of 200 MPa/5 min is sufficient to assess changes in gene expression. A pressure of 400 MPa/5 min is commonly used in the industry [33] and was chosen in order to compare the direction of change between the two pressure parameters. After HPP treatment at 400 MPa for 5 min, the strains were regenerated following the methodology provided by Valdramidis et al. [34] with minor modifications. Regeneration after 400 MPa/5 min was applied, as the pressure used causes the cells to transition to a physiological state, preventing their direct assessment using culture methods [25]. Briefly, after HPP, 1 mL of each culture was inoculated into fresh TSB (Merck, Darmstadt, Germany) and incubated at 30 °C until growth was observed. Cultures were then streaked onto ALOA (Agar Listeria Ottavani & Agosti; Merck, Darmstadt, Germany) agar plates (in triplicate) and incubated at 37 °C for 48 h to confirm species affiliation of regenerated strains.

### 4.3. Gene Expression

Total RNA was extracted using the Total RNA Mini Plus kit (A&A Biotechnology, Gdynia, Poland). For the control group, RNA was isolated from overnight cultures, whereas for strains treated at 200 MPa/5 min, RNA was extracted immediately after HPP treatment. For strains exposed to 400 MPa/5 min, RNA was obtained from regenerated cells in order to obtain RNA of sufficient quality and quantity for gene expression analysis. RNA samples were preserved in phenol (A&A Biotechnology, Gdynia, Poland) at −80 °C. Following isolation, RNA was purified and concentrated using the CleanUp RNA Concentrator kit (A&A Biotechnology, Gdynia, Poland) according to the manufacturer’s instructions. RNA integrity assessment and cDNA synthesis were performed as previously described by Adamski et al., 2025 [32]. Briefly, RNA integrity and purity were assessed by agarose gel electrophoresis (1.2% agarose, 0.5× TBE, 90 V, 1 h) and spectrophotometry (260/280 nm). RNA was then reverse transcribed into cDNA using the TranScriba kit (A&A Biotechnology, Gdynia, Poland) with MMLV reverse transcriptase, random hexamers, and RNase inhibitor, following the manufacturer’s instructions.

Expression of nine virulence genes associated with biofilm formation (*flaA*, *agrA*, *agrC*, *actA*, *prfA*, *hly*, *inlB*, *sigB*, *degU*) was assessed by qPCR in three independent biological replicates using SYBR Green detection on the Rotor-Gene Q system (Qiagen, Montreal, QC, Canada), following the previously described protocol [32]. Each 10 μL reaction contained 5 μL of PowerUp SYBR Green Master Mix (Thermo Fisher Scientific, Waltham, MA, USA), 1 μL of each primer (800 nM), and 1 μL of cDNA. Negative controls without template (NTC) and no-reverse transcription controls were included in each RT-qPCR run to exclude reagent contamination and to verify the absence of genomic DNA amplification, respectively. Cycling conditions were 50 °C for 2 min, 95 °C for 10 min, followed by 40 cycles of 95 °C for 15 s and gene-specific annealing for 60 s. A melting curve from 60 to 95 °C in 0.5 °C increments was used to confirm specificity. Gene expression was normalized to 16S rRNA [24], quantified using the Pfaffl method [35], and thresholds were determined with Rotor-Gene Q-Series Software 2.3.5. Expression ratios ≥ 2 or ≤0.5 were considered significant over- or underexpression, respectively. Primer sequences used in this study are listed in Table S1 [36,37,38,39,40,41].

### 4.4. Biofilm Viability

Prior to initiating biofilm development, the coupons were disinfected by immersion in 70% (*v*/*v*) ethanol for 10 min, rinsed twice with sterile distilled water, and subsequently left to dry under laminar flow conditions for 2 h. Afterwards, they were sterilized by autoclaving at 121 °C for 15 min [42]. Quantification of viable bacteria within biofilms was performed as described by Panebianco et al. [43] with minor modifications. Overnight *L. monocytogenes* cultures were grown in TSB (Merck, Darmstadt, Germany), centrifuged at 4000 rpm for 10 min, and washed three times with PBS (pH 7.3 ± 0.2). The bacterial suspensions were standardized to an OD550 of 0.125 (~8 log CFU/mL) in fresh TSB. Then, 3 mL of each suspension was applied to 12-well polystyrene plates containing biofilm coupons made of stainless-steel 304 (SS304), polyethylene terephthalate (PET), or polypropylene (PP) (BioSurface Technologies Corp., Bozeman, MT, USA) and incubated at 25 °C for 72 and 168 h, replacing 20% of the medium every 24 h with fresh TSB using a sterile serological pipette to maintain nutrient availability and prevent the accumulation of metabolic waste, thereby supporting sustained biofilm growth. After incubation, biofilm-coated coupons were aseptically transferred into 3 mL PBS and subjected to three cycles of sonication (1 min; 38 kHz) and vortexing (30 s) to detach the biofilm. Suspensions were plated on Oxford agar (Merck, Darmstadt, Germany) in triplicate and incubated at 37 ± 2 °C for 48 h. Bacterial counts were expressed as log(CFU/cm^2^). Cultures of the tested *L. monocytogenes* strains not subjected to pressure served as controls.

### 4.5. Biofilm Biomass

Biofilm production indices (BPIs) were determined following the protocol of Di Bonaventura et al. [44] with minor modifications. Overnight *L. monocytogenes* cultures (prepared as in Section 4.4) were grown in 12-well polystyrene plates containing 3 mL of TSB and coupons made of SS304, PET, or PP. After incubation (25 °C/24 h), coupons were aseptically transferred to new 12-well plates using sterile tweezers gently and rinsed three times with sterile PBS to remove non-adherent cells, while control samples were left uninoculated. Biofilms were fixed by drying the coupons at 60 °C for 1 h and then stained with 3 mL of 2% crystal violet (Hurt-Chem, Duchnice, Poland) for 20 min. Excess dye was removed by washing three times with distilled water. To quantify biofilm biomass, 3 mL of 33% acetic acid was added to solubilize the bound dye, and 200 µL of the resulting solution was transferred to a microtiter plate for absorbance measurement at 595 nm. The cultures of the tested *L. monocytogenes* strains, which were not subjected to pressure at that time, served as positive controls. For each material type (SS304, PET, PP), uninoculated coupons were included as negative controls. Biofilm production indices were calculated using the following formula:

$$BPI=\left(\frac{ODmean}{surface\;area\;in\;{mm}^{2}}\right)\times 1000$$

where ODmean represents the mean value from three independent biological replicates.

### 4.6. Statistics

All experiments were carried out in three experimental replicates. Data are presented as arithmetic means ± standard deviations. Statistical analyses were performed using Statistica 13.0 (StatSoft, Hamburg, Germany), with significance assessed by Kruskal–Wallis or ANOVA followed by Tukey HSD post hoc test at *p* ≤ 0.05. Graphs were generated using Python (version 3.10) in the Google Colab environment (Google LLC, Mountain View, CA, USA) using Matplotlib 3.11.0 and Seaborn libraries 0.13.2.

## 5. Conclusions

HPP significantly reduced biofilm viability and biomass of *L. monocytogenes* on all tested surfaces in a pressure-dependent manner. Transcriptomic analysis revealed a significant modulation of several genes associated with biofilm formation. These findings demonstrate that HPP significantly affects both phenotypic and genotypic traits linked to persistence, providing insights into how *L. monocytogenes* adapts to sublethal high-pressure stress in food-processing environments. However, further studies integrating transcriptomic approaches are required to elucidate the regulatory networks underlying the observed pressure-dependent responses of *L. monocytogenes*.

## Figures and Tables

**Table 1:** Mean of viable cell counts [log_10_(CFU/cm^2^)] ± SD for *L. monocytogenes* strains (*n* = 6) on different surface types (SS304, PET, PP) after 72 h and 168 h of incubation.

Material	Incubation Period [h]	
	72 h	168 h
	Control	
**SS304**	7.05 ± 0.23	6.99 ± 0.18
**PP**	7.97 ± 0.32	7.01 ± 0.21
**PET**	7.78 ± 0.18	6.60 ± 0.30
	HPP—200 MPa/5 min	
**SS304**	7.28 ± 0.20	5.98 ± 0.18 *
**PP**	6.81 ± 0.43 *	6.68 ± 0.20 *
**PET**	6.54 ± 0.04 *	6.54 ± 0.04 *
	HPP—400 MPa/5 min	
**SS304**	5.77 ± 0.25 *	5.51 ± 0.28 *
**PP**	6.29 ± 0.20 *	5.64 ± 0.20 *
**PET**	6.07 ± 0.05 *	5.84 ± 0.09 *

* A statistically significant reduction compared to the control group (T: Kruskal–Wallis; *p* < 0.05). Abbreviations: SS304—stainless-steel 304; PP—polypropylene; PET—polyethylene terephthalate.

## Data Availability

The original contributions presented in this study are included in the article/Supplementary Materials. Further inquiries can be directed to the corresponding authors.

## Supplementary Materials

The following supporting information can be downloaded at: https://www.mdpi.com/article/10.3390/ijms27125366/s1.

## Abbreviations

The following abbreviations are used in this manuscript:

ALOA	Agar Listeria Ottavani and Agosti
ANOVA	Analysis of Variance
BFC	Biofilm Forming Capacity
BPI	Biofilm Production Index
cDNA	Complementary DNA
CFUs	Colony Forming Units
CFU/cm^2^	Colony Forming Units per Square Centimeter
ECDC	European Centre for Disease Prevention and Control
EFSA	European Food Safety Authority
EPSs	Extracellular Polymeric Substances
EU	European Union
HPP	High Pressure Processing
HSD	Honestly Significant Difference
MPa	Megapascal
NCBI	National Center for Biotechnology Information
NTC	No Template Control
OD	Optical Density
PBS	Phosphate Buffered Saline
PET	Polyethylene Terephthalate
PP	Polypropylene
qPCR	Quantitative Polymerase Chain Reaction
RNA	Ribonucleic Acid
rRNA	Ribosomal Ribonucleic Acid
RT-qPCR	Reverse Transcription Quantitative Polymerase Chain Reaction
SD	Standard Deviation
SS304	Stainless-Steel 304
TSA	Tryptic Soy Agar
TSB	Tryptic Soy Broth
