# Supplementary material for: High-Pressure Processing Alters Biofilm Persistence and Virulence Gene Expression in Listeria monocytogenes Strains
Source: Int J Mol Sci. 2026 Jun 14;27(12):5366. doi: 10.3390/ijms27125366 (PMC13299948; doi:10.3390/ijms27125366)
Supplement: Supplementary file 1 [file ijms-27-05366-s001.zip › ijms-4239046-supplementary.pdf]

Table S1. Primers used in study.

Primer	Sequence 5'-3'	Reference
**rt-actA-F**	ACAACGGTGAGCAAACAGGA	[41]
**rt-actA-R**	CGCTCCACTTGTAGAGTTGGT	
**rt-hly-F**	TGCCAGGTAACGCGAGAAAT	
**rt-hly-R**	TGGTGCCCCAGATGGAGATA	
**rt-plcA-F**	CCCATTAGGCGGGAAAGCAT	
**rt-plcB-R**	ACCTGCCAAAGTTTGCTGTG	
**rt-prfA-F**	TTAGCGAGAACGGGACCATC	
**rt-prfA-R**	TAACGTATGCGGTAGCCTGC	
**rt-flaA-R**	ATTGACGCATACGTTGCAAGAT	[39]
**rt-flaA-F**	GACTTGTTACAAACAGCGGATTCA	
**rt-agrA-R**	AACCACGCGGATCAAACTTC	[37]
**rt-agrA-F**	GCAGCCGGAACATGAATGG	
**rt-agrC-R**	CGCGATTCGAATAACTGGATTT	[36]
**rt-agrC-F**	TATTTTGCTAGATAATGCGGTTGAA	
**rt-prfA-R**	TAACGTATGCGGTAGCCTGC	[40]
**rt-prfA-F**	TTAGCGAGAACGGGACCATC	
**rt-inlB-R**	ACATAGCCTTGTTTGGTCGG	[38]
**rt-inlB-F**	AAAGCACGACGATTTCATGGGAG	
**rt-sigB-R**	TGACGTTGGATTCTAGACAC	[37]
**rt-sigB-F**	TCATCGGTGTCACGGAAGAA	
**rt-degU-R**	CCCAATTCCGCGGTTACTT	[39]
**rt-degU-F**	ACGCATAGAGAGTGCGAGGTATT	

Table S2. Viable cell counts [$log_{10}(CFU/cm^2)$] ± SD for *L. monocytogenes* strains (n = 6) on different surface types (SS304, PET, PP) after 72 h and 168 h of incubation.

Strain	Surface	Control 72h	Control 168h	HPP 200-MPa 72h	HPP 200-MPa 168h	HPP 400-MPa 72h	HPP 400-MPa 168h
**LM8**	SS304	6.88 ± 0.11	7.13 ± 0.12	7.48 ± 0.10	6.15 ± 0.15	5.61 ± 0.04	5.40 ± 0.06
**LM14**	SS305	6.78 ± 0.22	6.88 ± 0.10	7.18 ± 0.06	5.94 ± 0.22	5.45 ± 0.19	5.23 ± 0.18
**LM40**	SS306	7.13 ± 0.18	6.83 ± 0.16	7.54 ± 0.17	6.01 ± 0.06	5.63 ± 0.17	5.35 ± 0.18
**LM41**	SS307	7.32 ± 0.15	7.26 ± 0.07	7.18 ± 0.12	5.83 ± 0.08	6.01 ± 0.18	5.90 ± 0.15
**LM47**	SS308	6.83 ± 0.07	7.03 ± 0.10	7.0 ± 0.06	5.73 ± 0.05	5.83 ± 0.19	5.35 ± 0.19
**LM48**	SS309	7.34 ± 0.07	6.83 ± 0.11	7.3 ± 0.13	6.21 ± 0.10	6.11 ± 0.05	5.82 ± 0.13
**LM8**	PET	7.82 ± 0.05	7.26 ± 0.13	7.17 ± 0.05	6.83 ± 0.11	6.13 ± 0.11	5.33 ± 0.14
**LM14**	PET	8.40 ± 0.20	7.19 ± 0.19	6.73 ± 0.21	6.73 ± 0.09	6.33 ± 0.06	5.76 ± 0.12
**LM40**	PET	7.58 ± 0.15	6.97 ± 0.08	7.13 ± 0.09	6.43 ± 0.2	6.12 ± 0.20	5.77 ± 0.04
**LM41**	PET	7.81 ± 0.17	6.67 ± 0.14	7.19 ± 0.17	6.83 ± 0.11	6.54 ± 0.16	5.67 ± 0.06
**LM47**	PET	7.88 ± 0.04	6.91 ± 0.15	6.13 ± 0.10	6.43 ± 0.09	6.11 ± 0.10	5.45 ± 0.05
**LM48**	PET	8.32 ± 0.22	7.08 ± 0.05	6.53 ± 0.14	6.83 ± 0.14	6.52 ± 0.05	5.83 ± 0.16
**LM8**	PP	7.88 ± 0.20	6.73 ± 0.16	6.51 ± 0.14	5.00 ± 0.07	6.05 ± 0.10	5.85 ± 0.10

**LM14**	PP	7.47 ± 0.08	6.13 ± 0.07	6.59 ± 0.08	6.04 ± 0.19	6.0 ± 0.10	5.70 ± 0.14
**LM40**	PP	7.84 ± 0.07	6.67 ± 0.05	6.55 ± 0.22	5.48 ± 0.05	6.10 ± 0.18	5.90 ± 0.21
**LM41**	PP	7.88 ± 0.07	6.43 ± 0.22	6.53 ± 0.19	5.30 ± 0.23	6.05 ± 0.16	5.80 ± 0.09
**LM47**	PP	7.94 ± 0.10	7.03 ± 0.22	6.56 ± 0.22	6.02 ± 0.19	6.15 ± 0.21	5.95 ± 0.12
**LM48**	PP	7.67 ± 0.14	6.61 ± 0.19	6.49 ± 0.21	6.13 ± 0.08	6.05 ± 0.13	5.85 ± 0.18

Abbreviations: SS304 – stainless steel 304; PP – polypropylene; PET - polyethylene terephthalate

Table S3. Combined statistical summary for biofilm biomass and viable cell counts.

Variable	Comparison	Surface	Time	Shapiro–Wilk (W, p)	Levene p	Test	Statistic	p-value	**Post-hoc** (Tukey HSD)
**BPI**	Control	SS304 vs PET vs PP	—	SS304: 0.947, 0.704; PET: 0.918, 0.512; PP: 0.933, 0.618	0.192	ANOVA	F = 42.8	p < 0.000001	SS304 > PET, SS304 > PP
**BPI**	200 MPa	SS304 vs PET vs PP	—	SS304: 0.953, 0.742; PET: 0.926, 0.566; PP: 0.948, 0.711	0.411	ANOVA	F = 9.12	0.0013	SS304 > PET, SS304 > PP
**BPI**	400 MPa	SS304 vs PET vs PP	—	SS304: 0.941, 0.668; PET: 0.903, 0.441; PP: 0.957, 0.771	0.335	ANOVA	F = 3.48	0.0482	no significant differences
**CFU**	IIa vs IVb	SS304	72 h	0.846, 0.041	—	Kruskal–Wallis	H = 1.12	0.290	—
**CFU**	IIa vs IVb	SS304	168 h	0.882, 0.072	—	Kruskal–Wallis	H = 2.03	0.154	—

**CFU**	IIa vs IVb	PET	72 h	0.801, 0.018	—	Kruskal–Wallis	H = 3.87	0.049	—
**CFU**	IIa vs IVb	PET	168 h	0.825, 0.027	—	Kruskal–Wallis	H = 5.42	0.020	—
**CFU**	IIa vs IVb	PP	72 h	0.858, 0.049	—	Kruskal–Wallis	H = 2.76	0.096	—
**CFU**	IIa vs IVb	PP	168 h	0.812, 0.022	—	Kruskal–Wallis	H = 6.18	0.013	—

Bibliography:

36. Fan, Q.; Zhang, Y.; Yang, H.; Wu, Q.; Shi, C.; Zhang, C.; Wang, X. Effect of coenzyme Q0 on biofilm formation and attachment-invasion efficiency of *Listeria monocytogenes*. *Food Control* **2018**, *90*, 274–281. https://doi.org/10.1016/j.foodcont.2018.02.047
37. Yan, H.; Wu, M.; Gao, B.; Bu, X.; Dong, Q.; Hirata, T.; Li, Z. Inhibition and eradication of *Listeria monocytogenes* biofilm using the combined treatment with nisin and sesamol. *LWT* **2024**, *198*, 116015. https://doi.org/10.1016/j.lwt.2024.116015
38. Oh, H.; Kim, S.; Lee, S.; Lee, H.; Ha, J.; Lee, J.; Yoon, Y. Prevalence, serotype diversity, genotype and antibiotic resistance of *Listeria monocytogenes* isolated from carcasses and humans in Korea. *Korean J. Food Sci. Anim. Resour.* **2018**, *38*, 851. https://doi.org/10.5851/kosfa.2018.e5
39. Upadhyay, A.; Upadhyaya, I.; Kollanoor-Johny, A.; Venkitanarayanan, K. Antibiofilm effect of plant derived antimicrobials on *Listeria monocytogenes*. *Food Microbiol.* **2013**, *36*, 79–89. https://doi.org/10.1016/j.fm.2013.04.010
40. Wiśniewski, P.; Chajęcka-Wierzchowska, W.; Zadernowska, A. High-pressure processing—impacts on the virulence and antibiotic resistance of *Listeria monocytogenes* isolated from food and food processing environments. *Foods* **2023**, *12*, 3899. https://doi.org/10.3390/foods12213899
41. Zakrzewski, A.; Gajewska, J.; Chajęcka-Wierzchowska, W.; Zadernowska, A. Effect of sous-vide processing of fish on the virulence and antibiotic resistance of *Listeria monocytogenes*. *NFS J.* **2023**, *31*, 155–161. https://doi.org/10.1016/j.nfs.2023.05.003
